# CO Oxidation at 20 °C on Au Catalysts Supported on Mesoporous Silica: Effects of Support Structural Properties and Modifiers

**DOI:** 10.3390/ma11060948

**Published:** 2018-06-04

**Authors:** Abigail Moreno-Martell, Barbara Pawelec, Rufino Nava, Noelia Mota, Luis Escamilla-Perea, Rufino M. Navarro, Jose L.G. Fierro

**Affiliations:** 1 Division of Research and Postgraduate Studies, Faculty of Engineering, Universidad Autónoma de Querétaro (UAQ), Cerro de las Campanas s/n, Querétaro 76010, Mexico; morenomartell@gmail.com (A.M.-M.); luisep25@hotmail.com (L.E.-P.); 2 Instituto de Catálisis y Petroleoquímica, CSIC, Marie Curie 2, Cantoblanco, 28049 Madrid, Spain; noelia.mota@icp.csic.es (N.M.); r.navarro@icp.csic.es (R.M.N.); jlgfierro@icp.csic.es (J.L.G.F.)

**Keywords:** gold nanoparticles, SBA-16, SBA-15, HMS, DMS-1, CO oxidation

## Abstract

In this work we report the effects of support structural properties and its modification with some metal oxides modifiers on the catalytic behavior of Au catalysts in the total CO oxidation at 20 °C. Au catalysts were supported on mesoporous silica materials (MSM) having different structural properties: Channel-like (SBA-15), cage-like (SBA-16), hexagonal (HMS), and disordered (DMS-1) structures. The effect of the modifier was evaluated by comparison of the catalytic response of the SBA-15-based catalysts modified with MgO, Fe_2_O_3_, TiO_2_, and CeO_2_. The chemical, structural, and electronic properties of the catalysts were investigated by a variety of techniques (metal content analysis by ICP-OES, N_2_ physisorption, XRD, UV-vis DRS, DRIFTS of adsorbed CO and OH regions, oxygen storage capacity (OSC), HR-TEM, and XPS). The activity of calcined catalysts in the CO oxidation reaction were evaluated at steady state conditions, at 20 °C, atmospheric pressure, and when using, as feed, a 1%CO/1%O_2_/98% gas mixture. The work clearly demonstrated that all Au catalysts supported on the mesoporous silicas modified with metal oxides were more active than the Au/SBA-15 and Au/MgO reference ones. The support structural properties and type of dopant were important factors influencing on the catalyst behavior. Concerning the support textural properties, it was found that the HMS substrate with the wormhole-structure offers better porosity and specific surface area than their silica counterparts having channel-like (SBA-15), cage-like (SBA-16), and disordered (DMS-1) mesoporous structures. Concerning the effect of modifier, the best catalytic response was achieved with the catalysts modified with MgO. After activation by calcination at 200 °C for 4 h, the Au/MgO/HMS catalyst exhibited the best catalytic performance, which was ascribed to the combined effects of the best structural properties, a large support oxygen storage capacity and homogeneous distribution of gold particles on the support (external and inner). Implications of the type of active sites (Au^1+^ or Au^0^), support structural properties and role of modifier on the catalytic activity are discussed.

## 1. Introduction

Since the discovery of the high activity of extremely fine gold nanoparticles in a low temperature CO oxidation [1,2], the use of Au/oxide and inverse oxide/Au catalysts in different reactions have been extensively studied [3,4,5,6,7,8,9,10,11,12,13,14,15,16,17,18,19,20,21,22,23,24,25,26]. Many recent reviews on those catalytic systems evidenced that the catalytic mechanism of the supported gold catalysts in the CO oxidation reaction is complicated due to the various combined factors influencing on the catalytic response of Au-based catalysts, such as availability of low-coordinated sites on the surface of very small particles [26], nature of support material (its chemical composition and structure) [11,12,13,14,15,16,17,18], catalyst preparation procedure [19,20,21], reaction conditions, etc. In addition, there is general consensus that the most important effect is related to the availability of low-coordinated sites on the surface of very small gold particles [26]. However, it is still not clear how the complex nature of the interface between Au and support may influence on the nature of active sites in the CO oxidation reaction [23,24]. The recent advances in the development of gold catalysts for CO oxidation are presented in a few excellent revisions [11,23,25].

Concerning the support substrates, the Au catalysts supported on the transition metal oxides, such as Fe_2_O_3_, CeO_2_, or TiO_2_, were the most extensively investigated because of their direct participation in the CO oxidation reaction mechanism linked with their ability to adsorb or store oxygen at low temperatures, lattice oxygen mobility, stabilization of the Au nanoparticles by inducing a stronger metal-support interaction, and/or the creation of ionic gold species [21]. Unfortunately, Au catalysts supported on reducible oxides exhibit lower specific surface areas than their counterparts supported on mixed oxides [18]. On the contrary to metal oxides with variable valence states (Fe_2_O_3_, CeO_2_, or TiO_2_, etc.), the SiO_2_, Al_2_O_3_, or MgO substrates are relatively difficult to be reduced. As a consequence, they are generally considered to be “inert” supports, offering a relatively weak metal-support interaction. However, the gold nanoparticles could be located on the surface of the irreducible metal oxides with F centers or defects [11]. In this sense, the defect sites on MgO (i.e., oxygen deficiency) were demonstrated to be essential for trapping the diffusing Au atoms or clusters on the surface [22]. 

Recently, there is great interest for developing gold catalysts supported on ordered mesoporous silica materials (MSM), such as MCM-48, HMS, or SBA-15 [7,8,9,14,15,16,17,26,27]. This is because of their interesting textural properties, such as high specific surface area, large pore diameter, and ordered pore structure In particular, the latter factor should be important for optimization of the catalyst performance because it allows trapping of Au particles within inner support porous structure [25]. In this sense, new designs of the Au-based catalysts showing core-shell, yolk-shell, or dumbbell nanostructures have been reported [23,24]. The modification of the MSM materials, such as MCM-48, HMS, or SBA-15, by incorporation of transition metal oxides led to change of the isoelectric point of the silica, allowing the incorporation of the gold nanoparticles by the simple deposition-precipitation method [25]. 

A detailed survey of recent developments on gold catalysts supported on ordered mesoporous silica materials by Gutiérrez et al. [25] indicated that there are few studies on the influence of their support structural properties on the catalytic behavior. This prompts us to study the effect of support structural properties on the activity of gold catalysts in low temperature CO oxidation. With this aim, four mesoporous silicas with different morphologies were synthetized and used for supporting gold catalysts: (i) SBA-15 having hexagonal pores in a 2D array and long 1D channels (*p6mm* plane group) [27,28]; (ii) SBA-16 with a three dimensional cage-like (*Im3m*) structure and interconnected micro- and mesopores [28,29,30]; (iii) HMS showing hexagonal array of uniform pores forming wormhole structure assembled from a long alkyl chain neutral dodecylamine used as the surfactant [16], and (iv) disordered mesoporous silica (DMS-1) [31]. To the best our knowledge, there are no works comparing the catalytic activities of Au-based catalysts supported on all these substrates. 

In one series of catalysts, the mesoporous silicas (HMS, DMS-1, SBA-15, and SBA-16) were modified with nanoparticles of MgO (5 wt %) forming MgO/silica substrates. Because the isoelectric point of silica (IEP ~ 2.0) [11] increases upon MgO addition (IEP = 10.1), gold nanoparticles were easily deposited on the surface of these modified silicas by the simple deposition-precipitation method. On a first sight, the choice of MgO as an additive can be surprising because it is considered as an inert additive, which does not participate in oxygen activation. However, it has been reported that the deposition of gold nanoparticles on the surface of MgO allows for the obtainment of active catalytic systems in the CO oxidation reactions [4,32,33,34,35,36,37]. Taking into account that CO oxidation is strongly affected by the electronic state of the gold nanoparticles [38], one of the possible explanations given in literature is that the defect sites at the MgO surface might favor charge transfer from the surface to the gold cluster, thus, promoting the Au catalytic activity [39]. However, there are other factors which might contribute to enhance of activity, such as the supply of adsorption sites for oxygen that may migrate to the Au particles and the formation of a reactive gold-oxide interface at the particle perimeter [40]. The Au catalysts are usually prepared by dispersing Au particles on MgO support (conventional metal/oxide configuration) [35,36,37] or by deposition of MgO on Au(111) surface (inverse MgO/Au configuration) [4]. Recently, Rodriguez et al. [4] reported that the addition of small nanoparticles of MgO to Au(111) (inverse catalyst formulation) produced excellent catalyst for the oxidation of CO at room temperature. This was explained by authors as due to an enhancement in the strength of the MgO-Au interaction by using the inverse catalyst configuration [4]. Another interesting option of the catalyst formulation is the use of MgO as an additive. In this sense, the earlier works by Grisel and Nieuwenhuys [40,41] demonstrated that the addition of MgO to Al_2_O_3_ substrate stabilized gold nanoparticles against thermal sintering. However, the comparison of activities for different MgO-containing catalytic systems studied in literature is difficult if not impossible. This is because the activity of gold catalysts can be greatly influenced by gold particle size, the type of support, and reaction conditions [39]. 

Within this scenario, the objective of this work was to study the effect of support structural properties (SBA-15, SBA-16, HMS, and DMS-1) on the catalytic response of gold catalysts modified with MgO in the CO oxidation at 20 °C. In addition, the effect of the SBA-15 substrate modification with different types of metal oxides (MgO, Fe_2_O_3_, CeO_2_, and TiO_2_) was investigated. The catalysts were characterized by various techniques in an attempt to establish a relationship between catalyst structure and performance.

## 2. Experimental

### 2.1. Synthesis of Mesoporous Silica and Their Modification with Additives

The siliceous mesoporous SBA-15 material was synthesized according to the procedure described by Flodström and Alfredsson [42], whereas SBA-16 and DMS-1 were prepared according to the method proposed by Zhao et al. [43]. SBA-15 was prepared using Pluronic triblock copolymer (BASF, EO_20_-PO_70_-EO_20_, P123) as the structure-directing agent, whereas the preparation of DMS-1 and SBA-16 involved different concentrations of Pluronic F127 (BASF, EO_106_-PO_70_-EO_106_). In all cases, tetraethylorthosilicate (TEOS, purity 98%, Sigma-Aldrich, St. Luis, MO, USA) was used as a silica source. In a typical synthesis, the triblock copolymer was dissolved in a solution of water and HCl under stirring, and then the required amount of TEOS was added to the above solution at 35 °C and kept under stirring for 24 h. The solid obtained was then filtered, washed thoroughly with deionized water, and dried at 110 °C for 18 h. The organic template was removed by calcination at 500 °C for 6 h. 

The HMS molecular sieve was prepared at room temperature by the procedure reported by Tanev and Pinnavaia [44] using TEOS as the neutral silica precursor and dodecylamine (DDA, purity 98%, Sigma-Aldrich) as the neutral structure directing agent. Mesitylene (MES, purity 97%, Sigma-Aldrich, St. Louis, MO, USA) was used as a swelling agent. Briefly, the surfactant (DDA) and the corresponding amount of water were mixed under vigorous stirring to obtain a homogeneous solution. After MES addition to the surfactant solution, the surfactant-auxiliary solution was stirred for 15 min. Then, TEOS was added and the mixture was stirred at room temperature for about 20 h. As the pH increases during the synthesis, the pH of the gel was adjusted several times using acid in order to obtain hydrothermally stable mesoporous silica. After synthesis, the solid residue obtained was filtered, thoroughly washed with distilled water, and dried in air. Consequently, the sample was dried at 100 °C in air for 24 h and finally calcined at 540 °C for 6 h.

The MgO-modified supports were prepared by impregnating laboratory-synthetized SBA-15(16), HMS, and DMS-1 substrates with a solution of magnesium nitrate (Mg(NO_3_)_2_·6H_2_O; 99.999% purity, Sigma-Aldrich) of appropriate concentrations. After drying at 110 °C for 18 h, the calcination was carried out at 500 °C for 4 h. Similarly, CeO_2_/SBA-15 and TiO_2_/SBA-15 substrates were prepared at room temperature by impregnation of SBA-15 substrate with solutions of cerium nitrate (Ce(NO_3_)_3_·6H_2_O, 99.999%, Sigma-Aldrich) and titanium (IV) isopropoxide (IPOTi; 97% purity, Sigma-Aldrich, St. Luis, MO, USA) in 2-propanol (99.5% Sigma-Aldrich) of appropriate concentrations, respectively. The resulting solids were dried and calcined at the same conditions as those used for MgO-modified counterparts. 

### 2.2. Catalyst Preparation

The deposition-precipitation technique was employed to prepare the Au-loaded mesoporous silica catalysts. HAuCl_4_ (98%, Sigma-Aldrich, St. Luis, MO, USA) was used as source of the Au. To obtain the gold concentration corresponding to a theoretical Au loading of 3 wt %, the appropriate amount (50 mL) of an aqueous solution of 3 × 10^−3^ M of HAuCl_4_ was prepared. Then, the support (1 g) was dispersed in an aqueous solution of HAuCl_4_ at 70 °C for 1 h. In order to ensure the complete gold deposition, the pH of solution was adjusted to 11 with a solution 0.1 M of NaOH. In order to avoid the negative effect of chlorine ions on the catalyst activity [10], the solids obtained were several times washed with deionized water. Finally, the samples were dried overnight at 110 °C in air for 18 h. The catalysts obtained were: Au/SBA-15, Au/MgO/SBA-16; Au/MgO/SBA-15, Au/MgO/HMS, Au/MgO/DMS-1, Au/CeO_2_/SBA-15, Au/TiO_2_/SBA-15, and Au/Fe_2_O_3_/SBA-15. 

### 2.3. Catalyst Characterization

#### 2.3.1. N_2_ Adsorption-Desorption Isotherms

The textural properties of the naked supports and dried gold catalysts were determined using a Micromeritics TriStar 3000 apparatus (Micromeritics, Norcross, GA, USA) using nitrogen as an adsorbate. Prior to the experiments, the samples were degassed at 270 °C under vacuum for 5 h. The specific areas of the samples were calculated by applying the Brunauer-Emmett-Teller equation (BET) to the nitrogen adsorption data within the 0.005–0.25 *P*/*P_0_* range. In order to avoid the tensile strength (TSE) artefact, the pore size distribution (PSD) curves were calculated by applying the Brunauer-Emmett-Teller (BJH) method to the adsorption branches of the N_2_ isotherms. The total pore volume of the DMS-1 and SBA-5(16)-based systems was obtained from the isotherms at *P*/*P_0_* = 0.99. Since total pore volume cannot be evaluated for material exhibiting Type II isotherm [45], this value was not calculated for all the HMS-based systems. 

#### 2.3.2. Determination of Metal Content

The metal contents of the naked supports and dried gold catalysts were measured using inductively coupled plasma optical emission spectroscopy (ICP-OES Optima 3300 DV, Perkin Elmer, Waltham, MA, USA). The catalyst structural properties and the presence of crystallite phases were evaluated from low- and wide-angles powder X-ray diffraction patters, respectively. The assignment of the phases was based on the Joint Committee on Powder Diffraction Standards (JCPDS) cards. From the observed XRD line broadening, the average crystalline size was calculated using the Scherrer equation. The Au particle size was determined also from transmission electron microscopy (TEM) images using a JEOL FX 200 electron microscope (JEOL USA, Peabody) operated at 200 kV. Before analysis, the samples were crushed and dispersed in acetone and then spread on a holey carbon Cu microgrid. 

#### 2.3.3. Small-Angle X-ray Diffraction

Small-angle X-ray diffraction experiments of the as-prepared MgO-modified Au catalysts were performed on a Philips X’Pert spectrometer (Malvern Panalytical Ltd., Royston, UK) using CuKα (λ = 0.1504 nm) radiation. The XRD data were collected in the range 0.2–5° 2*θ* by using 0.02° step size and a counting time of 1.3 s per step.

#### 2.3.4. Powder X-ray Diffraction (XRD)

Wide-angle XRD experiments of the as-prepared catalysts were performed on a Philips X’Pert diffractometer (Malvern Panalytical Ltd., Royston, UK) equipped with a CuKα anode and a graphite monochromator. The XRD data were collected in the angular range 5–90° in 2*θ* using 0.05° step size and counting time of 10 s per step. The assignment of the crystalline phases was based on JCPDS powder diffraction cards provided with the software of a Philips X’Pert diffractometer (Malvern Panalytical Ltd., Royston, UK). 

#### 2.3.5. DRIFT Spectroscopy in the OH Region

The OH region of the as-prepared Au samples was studied by DRIFT spectroscopy using a JASCO FT/IR-6300 spectrophotometer (JASCO, Easton, WA, USA) equipped with a Harrick diffuse reflectance accessory (HVC-DRP cell, Harrick Scientific Products, Pleasentville, NY, USA). Before analysis, the samples were degassed in flowing He at 200 °C for 1 h. 

#### 2.3.6. DRIFT Spectroscopy of Adsorbed CO (DRIFTS-CO)

The DRIFT spectra of adsorbed CO were recorded with the same FTIR spectrophotometer (JASCO FT/IR-6300, Takyo, Japan) equipped with a Harrick diffuse reflectance accessory (HVC-DRP cell, Harrick Scientific Products, Pleasentville, NJ, USA). Before analysis, the as-prepared samples were pre-treated by passing dry air (99.999% purity; Air Liquide, Paris, France) at 200 °C for 1 h. After calcination, the samples were cooled under a flow of He and then treated at room temperature and ambient pressure by passing high purity 5%CO/Ar gases for 30 min. Before the entry to HVC-DRP cell, the gases were passed through the tramp cooled with liquid nitrogen. 

#### 2.3.7. Oxygen Storage Capacity (OSC)

The oxygen storage capacity of the dried pure supports was carried out in a Micromeritics TPD/TPR 2900 apparatus (Micromeritics, Norcross, GA, USA). After sample purging in a flow of He (50 mL min^−1^) at 250 °C for 0.5 h, the sample was cooled to room temperature. Then, the oxygen adsorption was performed at 30 °C for 30 min by passing 5 vol % O_2_/He gas mixture (50 mL·min^−1^ of total flow; Air Liquide), followed by TCD signal stabilization with He (50 mL·min^−1^) at the same temperature for 15 min. Finally, the OSC profiles were obtained by temperature-programmed desorption of adsorbed oxygen conducted by heating the sample from RT to 650 °C (10 °C min^−1^) under He flow. The amount of desorbed oxygen was determined on line with thermal conductivity detector (TCD, Micromeritics). 

#### 2.3.8. UV-vis Diffuse Reflectance Spectra (DRS UV-vis)

UV-Vis diffuse reflectance electronic spectra of the dried materials (in the 200–900 nm range) were recorded on a UV-vis-NIR Varian Cary 5000 spectrophotometer (Varian, Santa Clara, CA, USA) outfitted with a 150 mm diameter integrating sphere coated with Poly Tetra-Fluoro Ethylene (PTFE). The powder samples were mounted in a quartz cell, which provided a sample thickness greater than 3 mm and thus guaranteed “infinite” sample thickness. 

#### 2.3.9. X-ray Photoelectron Spectroscopy (XPS)

The X-ray photoelectron spectroscopy analysis of the dried samples were performed with a VG Escalab 200R spectrometer (Vacuum Generators, Crowborough, UK) equipped with a hemispherical electron analyzer (Vacuum Generators) and a Mg Kα (hν = 1253.6 eV) X-ray source (Vacuum Generators, UK). The samples were first mounted on a sample-rod in the pre-treatment chamber of the spectrometer and then outgassed at 130 °C for 1 h before transfer to the analysis chamber. The pressure in the analysis chamber during data collection was ~7 × 10^−9^ mbar. Survey spectra were measured at 200 eV pass energy, whereas for the individual peak energy regions, a pass energy of 50 eV was used. XPS spectra were processed using XPSPEAK software (Vacuum Generators, UK). Prior to the fitting procedure, a Shirley-type background was applied to the spectra, and a Gaussian/Lorentzian (90 G/10 L) functional line was applied. The Au 4f core excitations were deconvoluted into a minimal number of peaks taking into account that the Au 4f ionization process is characterized by the doublet of the two spin-orbit components (Au 4f_7/2_ at about 83.8 eV, and Au 4f_5/2_ at 87.0 eV) with a splitting of 3.2 eV. The intensity ratio *IAu* 4f_7/2_/*IAu* 4f_5/2_ was fixed to 1.5. Surface atomic ratios were calculated from the peak area ratios normalized by the corresponding atomic sensitivity factors provided by the software. 

#### 2.3.10. High Resolution Transmission Electron Microscopy (HRTEM)

The studies of the calcined Au/MgO/SBA-15 samples were carried out using a JEM 2100F microscope (JOEL, Peabody, MA, USA) operating with a 200 kV accelerating voltage and fitted with an INCA X-sight (Oxford Instruments, Abingdon, UK) energy dispersive X-ray microanalysis (EDX) system to verify the semi-quantitative composition of the supported phases. The fine powder of the ex-situ pre-treated (5% O_2_/He, 200 °C, 1 h) samples was dispersed ultrasonically in hexane at room temperature. A drop of the suspension was placed on lacey carbon-coated Cu grid. At least ten representative images were taken for each sample. More than 250 particles were measured in order to obtain statistically reliable data of the particle size. 

### 2.4. Catalytic Activity Measurements

The catalytic activity for CO oxidation under atmospheric pressure and 20 °C of reaction temperature was measured in a fixed-bed laboratory-scale flow reactor using ~40 mg of catalyst. Because the presence of water can potentially have a great influence on the reaction [10], prior to activity measurement, the catalyst was dried with N_2_ (99.999% of purity; Air Liquide) in order to remove H_2_O and other contaminants. For in situ calcination the dried catalyst were pre-treated in a flow of dried air (99.999% of purity; Air Liquide) at 200 °C for 4 h and cooled after in a flow of N_2_ to 20 °C. For activity measurements, high purity gases were introduced into the reactor with a total flow rate of 80 mL/min. The feed (CO/O_2_/N_2_ = 1/1/98 vol %) was purified by passing through a silica trap at dry-ice temperature. The molar flow rate of the CO in this gas mixture was 5.95 × 10^−7^ mole s^−1^. Activity tests were repeated several times in order to verify their reproducibility. The reacting gases and products were analyzed by Agilent Technologies 6890 N (Agilent Technologies, Santa Clara, CA, USA), Network System gas chromatograph. CO conversion was determined by analyzing the reactor exit gas stream by GC. CO conversion (*X_CO_*) and the specific reaction rates were calculated according to Equations (1) and (2), respectively:

(1) $$X_{CO}=\frac{{\left[CO\right]}_{in}-{\left[CO\right]}_{out}}{{\left[CO\right]}_{in}}\times 100,$$

where [*CO*]*_inlet_* and [*CO*]*_outlet_* are the inlet and outlet concentrations of CO, respectively.

*r* = *X_CO_* × *F_CO_*/*m_cat_*, (2)

where *r* is the specific reaction rate [mole/(g_cat_·s)], *X* is CO conversion, *F_CO_* is the molar flow rate of the CO (mole s^−1^), and *m_cat_* is the catalyst weight (g). The specific reaction rate was expressed also as moles of CO converted per gram of Au atom and per second, taking into account the gold loading of dried samples determined by ICP-OES technique. Assuming half-spherical shape of Au particles and 1.15 × 10^15^ Au atoms cm^−2^, the turnover frequencies (TOFs) values were calculated considering the mean diameter of Au particle (from TEM), Au loading (from ICP-OES), and CO conversion at reaction time of 10 min. 

## 3. Results

### 3.1. Catalyst Characterization

#### 3.1.1. Structural Characterization of Pure Supports and Fresh Catalysts

Metal loadings of naked supports and as-prepared gold catalysts (from ICP-OES) are summarized in Table 1 and Table 2, respectively. For the MO_x_/MSM substrates, M-atom loading was in the range 5.2–5.6 wt %. Only for the TiO_2_/SBA-15 substrate, the Ti content was a little higher (7.3 wt %). For the as-prepared Au/MgO/MSM catalysts, Au loading (in the range 0.5–0.9 wt %) was much lower than that of its dopant (5.2–5.6 wt % of Mg). As compared with MgO-modified catalyst, the other SBA-15-based catalysts modified with Fe_2_O_3_, TiO_2_, and CeO_2_ showed higher Au loading (2.5–2.9 wt % vs. 0.8 wt %). Both XPS and EDX/TEM data confirmed that all catalysts were Cl-free. 

**Figure 1:**
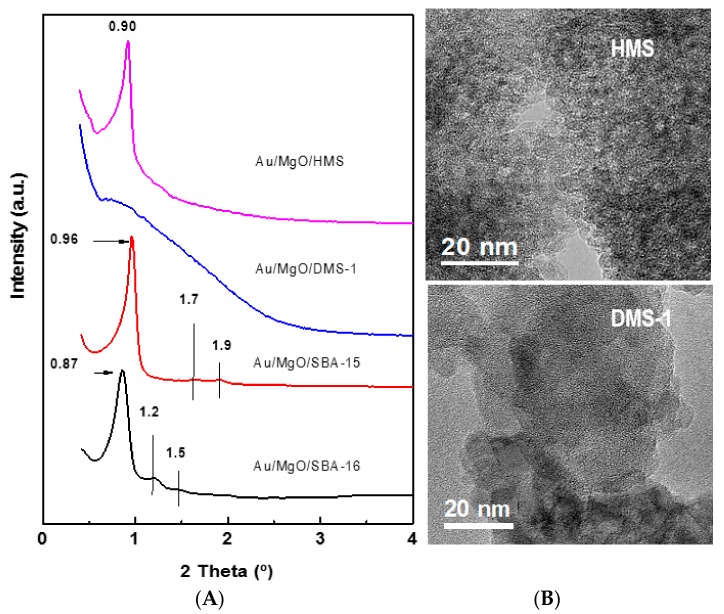
Low-angle XRD patterns of the as-prepared Au catalysts supported on different mesoporous silica substrates (**A**) and TEM images of the pure HMS and DMS-1 substrates showing wormhole and disordered porous support structures, respectively (**B**).

The structural properties of the as-prepared Au catalysts were studied using the small-angle XRD technique in the angular range 0.5–4° 2*θ* (Figure 1A). The XRD pattern of Au/MgO/SBA-16 sample exhibited a narrow peak at 2*θ* ~ 0.9°. According to body-centred packing of cubic symmetry of SBA-16, this reflection was indexed as [110] [29,30]. Two minor reflections at 2*θ* ~ 1.2° and 1.5° (indexed as [200] and [211]), associated with the cubic symmetry (*Im3m*) of cage-structured mesoporous SBA-16 silica, were also observed. Contrary to Au/MgO/SBA-16, the XRD pattern of Au/MgO/HMS catalyst exhibited only one peak at 2*θ* ~ 0.91°. The lack of higher-order diffraction peaks suggests the absence of long-range pore ordering [31]. Although higher order reflections were not observed, we assume that HMS substrate possessed a wormhole framework structure with local hexagonal symmetry [46]. As expected, Au/MgO/DMS-1 catalyst showed a broad low angle reflection in the 0.7–2.0 (°) 2*θ* range typical for the materials with disordered structure. In order to highlight the different textural characteristics of the HMS and DMS-1 materials in terms of absence of pore ordering, the HRTEM images of both materials are compared in Figure 1B. Finally, the Au/MgO/SBA-15 catalyst showed a very sharp diffraction peak at the 2*θ* of ~1° and two small peaks at the 2*θ* of 1.7° and 1.9°, which were indexed as the [1 0 0], [1 1 0], and [2 0 0] reflections, respectively. All those peaks were characteristics of the hexagonal space group (*p6mm*) symmetry. 

**Figure 2:**
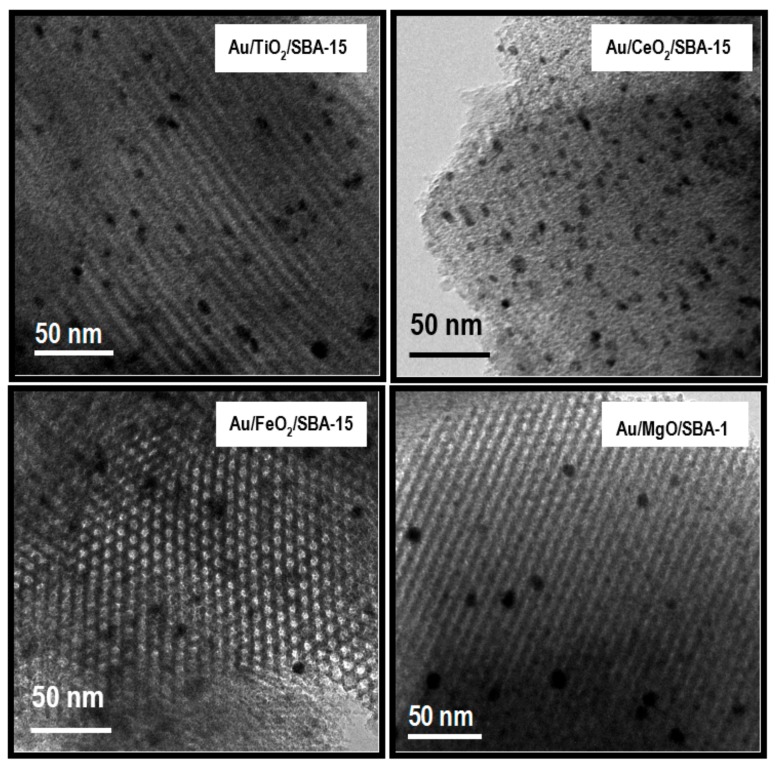
TEM micrographs showing parallel fringes (side-on view) of the calcined Au/TiO_2_/SBA-15 and Au/CeO_2_/SBA-15 samples, and hexagonal array (viewed along the pore direction) of the calcined Au/Fe_2_O_3_/SBA-15 and Au/MgO/SBA-15 catalysts.

**Figure 3:**
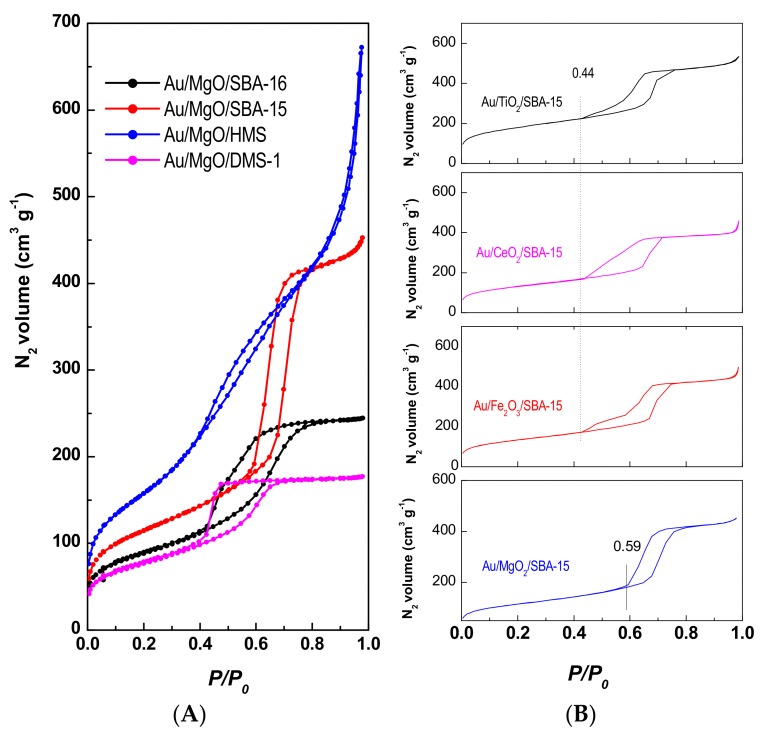
N_2_ adsorption-desorption isotherms at −196 °C of the as-prepared Au/MgO/MSM (**A**) and Au/MO_x_/SBA-15 (**B**) catalysts.

In good agreement with small-angle XRD, the TEM images of all SBA-15-based substrates evidenced the presence of a hexagonal system of lattice fringes along the pore direction, as shown in Figure 2 for the CeO_2_- and Fe_2_O_3_-modified sample. The side-on view of the long pores of SBA-15 substrate is shown in Figure 2 for TiO_2_- and MgO-modified catalysts. TEM images of all SBA-15-based catalysts (scale 50 nm) show small gold particles (mean particle size in range 5.5–7.5 nm). As expected from Au loading and particle size (Table 2), the density of the Au particles on the support surface was very different showing Au/MgO/SBA-15 the lowest density of Au particles on the support surface. From the combined XRD and HRTEM information, this can be explained considering the lower Au loading and larger average particle size of this sample (Table 2). For the Au/SBA-15 (TEM image not shown), the gold particles were irregularly distributed on the whole support. The sizes of gold particles range from 4 nm (inside pore system) to 20 nm (outside pore system). The bimodal size distribution of gold particles in the MO_x_-free Au/SBA-15 catalyst might suggest an Ostwald ripening mechanism [47], which operates via detachment and condensation of mobile Au or AuOx species. A similar bimodal size distribution was observed for Au catalysts supported on the acidic MnO_2_ substrate [48]. The average size of Au nanoparticles of the Au/SBA-15 catalyst was much larger than that of the Au catalysts supported on MO_x_ modified SBA-15 substrates (15 nm against 5.5–7.5 nm). The presence of large gold particles and the relative lower gold loading for Au/SBA-15 sample can be well explained by the highly acidic nature of SBA-15. In contrast, small and uniform gold nanoparticles were obtained on the Au/MO_x_/SBA-15 thanks to their higher point of zero charge (PZC) of the MO_x_/SBA-15 supported. Inspection of TEM images in Figure 3 shows that all SBA-15-based catalysts exhibit spherical- and oval-shaped gold particles. 

#### 3.1.2. N_2_ Adsorption-Desorption Isotherms

The textural properties of the as-prepared catalysts were evaluated by nitrogen adsorption-desorption isotherms at −196 °C. Figure 3A,B illustrate the N_2_ adsorption-desorption isotherms of as-prepared Au/MgO/MSM and Au/MO_x_/SBA-15 samples, respectively. According to a new IUPAC classification [45], Au/MgO/HMS sample showed Type II isotherm and hysteresis loop of type H3. The shape of this isotherm was characteristic of a material exhibiting a large amount of textural porosity, as confirmed by the hysteresis loop at high relative pressure (Figure 3A). In addition, the sharp increase of the N_2_ uptake in the range of *P*/*P_0_* = 0.2–0.4 in the isotherm of Au/MgO/HMS catalyst strongly suggests that this sample had a very high surface area. On the other hand, the Au/MgO/DMS-1 and Au/MgO/SBA-16 catalysts exhibited Type IV(a) isotherms characteristics of the materials having pores wider than ~4 nm. The hysteresis loop of the former sample was of type H2(a) whereas that of the latter was of type H2(b). Finally, the isotherm of Au/MgO/SBA-15 sample was of Type V; H1 hysteresis loop of this sample was typical of solids having a narrow range of uniform mesopores. Inspection of N_2_ isotherms in Figure 3B showed that the SBA-15-based samples exhibited Type V isotherms with H1 hysteresis loop. It was also quite clear that those catalysts exhibit some difference in the desorption path of their respective isotherms. Contrary to Au/MgO/SBA-15, the support modification with reducible metal oxides led to formation of ink-bottle shape pores [45]. 

The specific Brunauer-Emmett-Teller (BET) surface area, pore volume, and pore diameter of all supports and catalysts are listed in Table 1 and Table 2, respectively. As seen in Table 1, for the four synthetized pure silicas, the S_BET_ was high and decreased in the following order: HMS (976 m^2^ g^−1^) > SBA-15 (819 m^2^ g^−1^) > DMS-1 (772 m^2^ g^−1^) > SBA-16 (275 m^2^ g^−1^). As expected, the S_BET_ decreased after metal oxide incorporation due to pore occlusion of the mesoporous silica by gest molecules. Only the MgO/SBA-16 substrate showed similar S_BET_ as MgO-free SBA-16 (275 vs. 262 m^2^ g^−1^) suggesting that pore occlusion did not occur. This was confirmed by the NS_BET_ value of 1. 

Surprisingly, the Au-loaded samples showed a larger S_BET_ (Table 2) than the corresponding Au-free supports (Table 1). This was probably due to the extraction of guest molecules from the inner support porous structure during impregnation with Au precursor. The NS_BET_ values of the gold catalysts followed the trend: Au/MgO/DMS-1 (1.74) > Au/MgO/SBA-15 (1.41) > Au/MgO/SBA-16 (1.25) > Au/MgO/HMS (1.07). For the first three samples, the NS_BET_ values are much larger than 1 suggesting the main Au location on the support surface. The Au/MgO/HMS sample is unique showing this value close to 1. This means that similar amounts of gold nanoparticles are located within the silica porous structure and on the external support surface. On the contrary, the Au/SBA-15, Au/Fe_2_O_3_/SBA-15 and Au/CeO_2_/SBA-15 catalysts exhibited NS_BET_ much lower than 1, suggesting that some pores can be occluded by guest particles. 

The Au/MgO/HMS exhibited a high BET area and wide pore size distribution (not shown here). Taking into account that BET area did not change after Au loading by the deposition-precipitation method into MgO/HMS substrate, it was assumed that the original structure of HMS silica did not change. With the exception of Au/MgO/HMS catalyst, all other Au/MgO/silica catalysts showed Au crystallite sizes higher than that of the silica pore diameter. This means that the gold nanoparticles were not only located within the silica mesopores, but also on the external surface of the support, in line with those deduced from the NS_BET_ values (Table 2). Thus, the combined XRD and N_2_ adsorption-desorption data suggest that location of active phase depended on the support structural properties. Taking into account the importance of Au species location for the catalyst activity in CO oxidation reaction [11,12,13], the support structural properties should be the decisive factor influencing the final catalyst response in the target reaction. This point will be discussed here after.

Finally, regardless of the support structural properties, all gold catalysts exhibited an increase of the average pore diameter with respect to naked supports (Table 2). This can be probably linked with the formation of a new mesopores during Au deposition/precipitation on the support surface using an aqueous solution of gold acid (HAuCl_4_).

#### 3.1.3. Oxygen Storage Capacity (OSC) of Naked Supports Determined by TPD-O_2_

**Figure 4:**
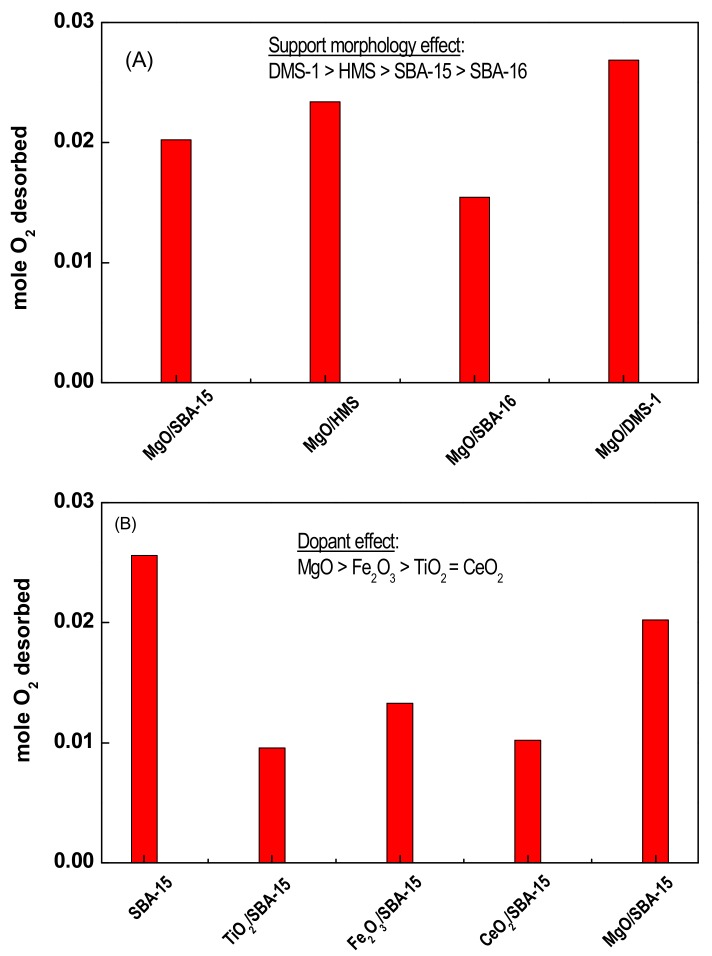
Oxygen storage capacity of the pure supports (from TPD-O_2_): Effects of support structural properties (**A**) and SBA-15 substrate modification with different dopants (**B**).

The possible relationship between oxygen storage capacity of the bare supports and the final catalyst activity was investigated by evaluation of their oxygen storage capacities (OSC). The oxygen storage capacity of all supports was measured at room temperature by oxygen flowing through the reactor. Figure 4A shows the influence of the support structural properties on the OSC values of the MgO-loaded mesoporous silica. As seen in this figure, the OSC values follow the trend: DMS-1 > HMS > SBA-15 > SBA-16. By comparison of the textural properties (Table 1) and OSC capacities (Figure 4A), it was clear that there was no correlation between the two properties. 

Concerning the effect of dopant (Figure 4B), the OSC values followed the order: SBA-15 > MgO/SBA-15 >> Fe_2_O_3_/SBA-15 > CeO_2_/SBA-15 > TiO_2_/SBA-15. On first sight, the low OSC values of the samples modified with reducible metal oxides seems to be surprising. However, one should to keep in mind that the information obtained from TPD-O_2_ measurements is different from that which can be obtained by comparison of the OSC capacity of naked reducible oxides. This is because the high redox potential of the reducible oxides is due to facile dynamics of oxygen exchange rather than due to the net capacity of such oxides to adsorb oxygen. In this work, the OSC results obtained over different mesoporous samples strongly suggested that the oxygen storage capacity depended on the support structural properties. The support modification with MO_x_ led to a decrease of the oxygen storage capacity due to the pore blocking by nonoparticles of metal oxides.

#### 3.1.4. Wide-Angle XRD Diffraction of Fresh Catalysts

**Figure 5:**
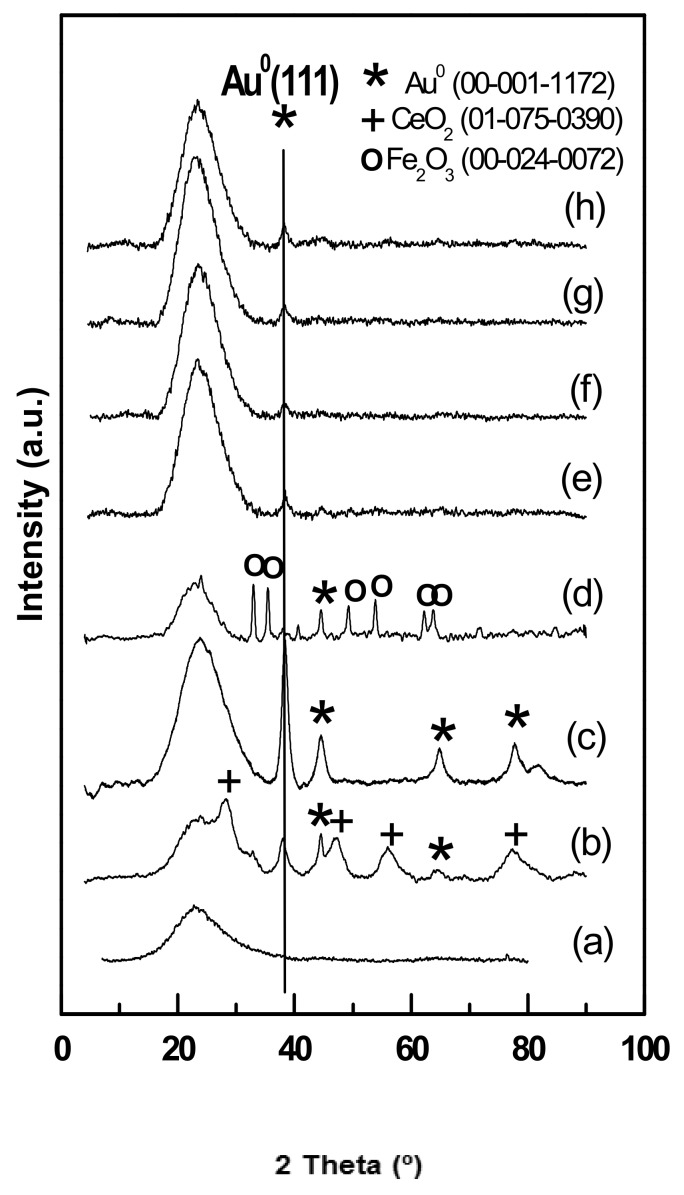
Wide-angle XRD patterns of the as-prepared Au samples supported on different mesoporous silica substrates: (**a**) Au/SBA-15; (**b**) Au/CeO_2_/SBA-15; (**c**) Au/TiO_2_/SBA-15; (**d**) AuFe_2_O_3_/SBA-15; (**e**) Au/MgO/SBA-16; (**f**) Au/MgO/SBA-15; (**g**) Au/MgO/HMS; (**h**) Au/MgO/DMS-1.

Wide-angle XRD technique was used to investigate the presence of any crystalline species in the supported gold catalysts. Figure 5 shows powder wide-angle XRD patterns of the dried Au catalysts supported on different carriers. As seen, regardless of the support structural properties and the type of dopant (MgO, CeO_2_, Fe_2_O_3_, TiO_2_), all the dried Au catalysts showed the wide line of amorphous silica centred at about 2*θ* of 24°. The Au catalysts supported on mesoporous silica modified with MgO did not exhibit the XRD peaks of MgO crystalline phase (JCPDS: 75-0447, 2*θ* = 36.8, 42.8, 62.2, 74.5 and 78.5°). This implies that MgO particles were amorphous and/or they are well dispersed on the support surface. The latter situation occurred when the size of MgO crystallites was below detection limit of XRD technique (<4 nm). Noticeably, the peaks characteristics of Mg_2_SiO_4_ silicate, where the cations Mg^2+^ were packed with (SiO_4_)^2-^ tetrahedra, as well as for tge MgSiO_3_ phase were not observed, also indicating that the solid-solid reaction between silica and magnesium oxide during calcination at 500 °C did not occur.

With the exception of the Au/MgO/HMS catalyst, all other Au/MgO/silica catalysts showed Au crystallite sizes higher than that of the silica pore diameter. This means that the gold nanoparticles were not only located within the silica mesopores, but also on the external surface of the support, in line with those deduced from the NS_BET_ values (Table 2). Taking into account the importance of Au species location for the catalyst activity in CO oxidation reaction [11,12,13], the support structural properties should be the decisive factor influencing on the final catalyst response in the target reaction. This point will be discussed here after.

Similar to the Au/MgO/HMS, the XRD pattern of Au/TiO_2_/SBA-15 catalyst did not exhibit peaks for its additive suggesting, its amorphous character or high dispersion of TiO_2_ crystallites on the support surface. On the contrary, the Au/Fe_2_O_3_/SBA-15 catalyst exhibited diffraction peaks centred at 2*θ* of 24.1°, 33.1°, 35.6°, 40.8, 49.3, and 54° ascribed to hexagonal rhombic-centred hematite (JCPDS 72-04669). The crystallite size calculated by the Scherrer equation, using the full width at half-height (FWHM) of the (104) diffraction peak of α-Fe_2_O_3_ phase, indicated that its crystalline size was about 16.7 nm (Table 2). Similarly, the Au/CeO_2_/SBA-15 catalyst exhibited diffraction peaks at 2*θ* of 28.5°, 33.1°, 47.5°, 56.3°, and 76.7°, which are characteristic of (111), (220) and (311) planes of cubic ceria structure (JCPDS-ICDD 01-075-0390). The calculation of the crystal size of the CeO_2_ phase by the Scherrer equation using the FWHM of the (111) peak revealed a crystal size of 4.8 nm. 

Regardless of type of dopant and support structural properties, all Au catalysts showed the only presence of metallic Au phase with the most intense peak at 2*θ* = 38.18° (JCPDS 00-004-0784). Based on the FWHM of the (111) diffraction peak of the Au^0^ phase, the particle size calculated by the Scherrer equation decreased in the order: Au/MgO/SBA-16 (7.9 nm) > Au/MgO/DMS-1 (7.4 nm) ≈ Au/MgO/SBA-15 (7.3 nm) ≈ Au/TiO_2_/SBA-15 (7.2 nm) > Au/MgO/HMS (6.3 nm) ≈ Au/CeO_2_/SBA-15 (6.1 nm). 

#### 3.1.5. UV-vis Diffuse Reflectance Spectroscopy (UV-vis DRS)

**Figure 6:**
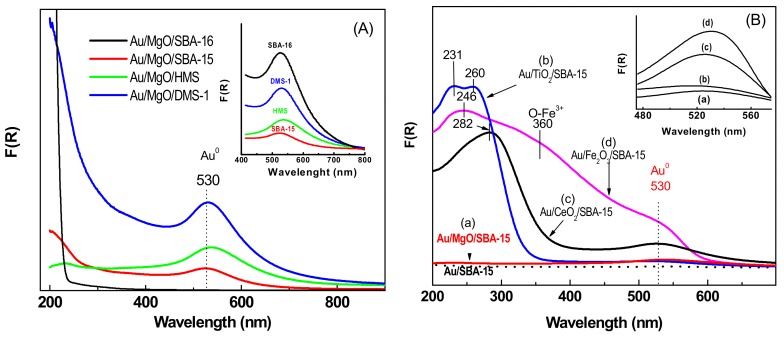
DRS UV-vis spectra of the as-prepared Au/MgO/MSM (**A**) and Au/MO_x_/SBA-15 (**B**) catalysts.

UV-vis diffuse reflectance spectroscopy was used to investigate valence state of Au in the dried samples. Figure 6A,B show the UV-vis spectra of the as-prepared Au/MgO/MSM and Au/MOx/SBA-15 samples, respectively. The inlets in both figures displayed the plasmon resonance band with maximum at ca. 530 nm suggesting the presence of Au-metal nanoparticles [49]. This band arises from the collective oscillations of the free conduction band electrons that are induced by incident electromagnetic radiation when the wavelength of the incident light greatly exceeds particle diameter [50]. Considering the effect of support structural properties, the intensity of the plasmon band followed the trend: Au/MgO/SBA-16 > Au/MgO/DMS-1 > Au/MgO/HMS > Au/MgO/SBA-15. As Au/MgO/SBA-16 catalysts showed the largest intensity of the Au surface plasmon resonance (SPR) among the catalysts studied. Unfortunately, an in-depth interpretation of the observed differences in the intensities of the plasmon bands to conclude on the mean Au particle size is impossible. This is because the mean particle diameter is not only related to the peak position and full width at half-height maximum (FWHM) of the plasmon band, but also depends on the dielectric properties of the supporting or surrounding metal oxide [49].

Concerning the metal oxide region of the dried Au/MO_x_/SBA-15 catalysts, the electronic spectra of the as-prepared Au/CeO_2_/SBA-15 catalyst showed one absorption band at about 282 nm due to oxygen-to-Ce^3+^ charge transfer processes [16]. This band exhibited a shadow at about 330 nm, which can be ascribed to the oxygen-to-Ce^4+^ charge transfer. On the other hand, the Au/Fe_2_O_3_/SBA-15 exhibited two important signals at about 357 and 246 nm commonly ascribed to oxygen-to-Fe^3+^ charge transfer [51]. Finally, Au/TiO_2_/SBA-15 catalyst showed two absorption bands at about 230 and 260 nm which can be assigned to Ti^4+^ sites octahedrally coordinated and highly dispersed TiO_2_ nanoparticles, respectively [52]. 

#### 3.1.6. DRIFTS Study of CO Adsorption

**Figure 7:**
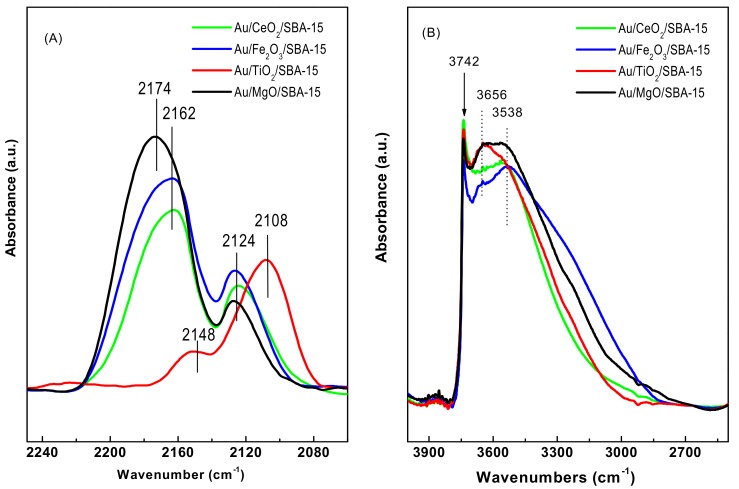
(**A**) DRIFT spectra of CO chemisorbed at RT and (**B**) DRIFT spectra showing the OH region of the pre-treated Au/Mo_x_/SBA-15 samples.

DRIFT spectroscopy of CO adsorption at room temperature was employed to study the mode of CO molecules adsorption on the surface of calcined catalyst. For in situ calcination, the dried catalyst was treated with an O_2_(20%)/He(80%) gas mixture at 200 °C for 1 h. The DRIFTS-CO spectra of the calcined Au/MO_x_/SBA-15 catalysts are shown in Figure 7A. The adsorption of CO on the surface of Au/TiO_2_/SBA-15 catalyst led to appearance of an intense band at 2108 cm^−1^ attributed to a single CO adsorption on step/kink sites of Au^0^ species [13]. Thus, in good agreement with XPS characterization (Table 3), the DRIFTS-CO spectrum of the Au/TiO_2_/SBA-15 sample suggested the only presence of metallic gold species. For this catalyst, the absence of the band at 2124 cm^−1^ allows us to conclude that the Au^+^ sites were not formed. This is an important conclusion of this study because it might explain the highest turnover frequency of this sample at the beginning of the reaction of CO oxidation. This point will be discussed below. As compared to the Au/TiO_2_/SBA-15, the MgO-, Fe_2_O_3_-, and CeO_2_-modified catalysts exhibited their first band blue-shifted to ~2124 cm^−1^ indicating CO adsorption on ionic Au sites [53]. 

For the Au/TiO_2_/SBA-15 catalyst, the second band appeared at around 2148 cm^−1^. Taking into account that CO adsorption on TiO_2_ rutile (2174 cm^−1^) and CO chemisorption on Ti^4+^ steps and terrace sites (2202 cm^−1^ and 2186 cm^−1^) [53] did not occur, it was more likely that the band at 2148 cm^−1^ was due to physically adsorbed CO [54]. For the Fe_2_O_3_- and CeO_2_-modified catalysts, the second band that appeared at about 2162 cm^−1^ can be ascribed to CO adsorption on the nanoparticles of corresponding dopant [13]. Indeed, the CO adsorption on pure CeO_2_ was reported to give rise to a band at 2168 cm^−1^ due to CO adsorbed on Ce^4+^ cations, being more or less coordinatively unsaturated (CUS sites) as compared to their co-ordination in the bulk [54]. Similarly, the CO adsorption on Mg^2+^ species gave rise to carbonyl band at 2174 cm^−1^ [55]. 

#### 3.1.7. DRIFT Spectra of OH Region of Fresh Catalysts

The hydroxylation region of the SBA-15-based samples was investigated by DRIFT spectroscopy of the OH region. Figure 7B shows the DRIFT spectra of the calcined SBA-15-based catalysts in the stretching vibration zone of OH groups (4000–3000 cm^−1^ spectral region). Although all spectra were similar, some differences could be discerned. The sharp band at about 3740 cm^−1^ was due to the stretching vibration mode of isolated OH groups (Si-OH) [56]. Other two broad bands at about 3650 and 3540 cm^−1^ can be due to stretching vibration mode of bridged hydroxyls and/or Si–OH groups interacting with the parent oxide [56]. Finally, a very broad band extending from 3500 to 3100 cm^−1^ is usually associated to the stretching vibration mode of OH groups in adsorbed molecular water [57]. The Au/MgO/SBA-15 sample exhibited largest intensity of the bands associated with bridging silanol groups, suggesting the presence of MgO species. Similarly, the appearance of bridged hydroxyls due to various Al species was observed after Al incorporation by post-synthesis method on the SBA-15 [56]. 

#### 3.1.8. X-ray Photoelectron Spectroscopy of Fresh Catalysts

**Figure 8:**
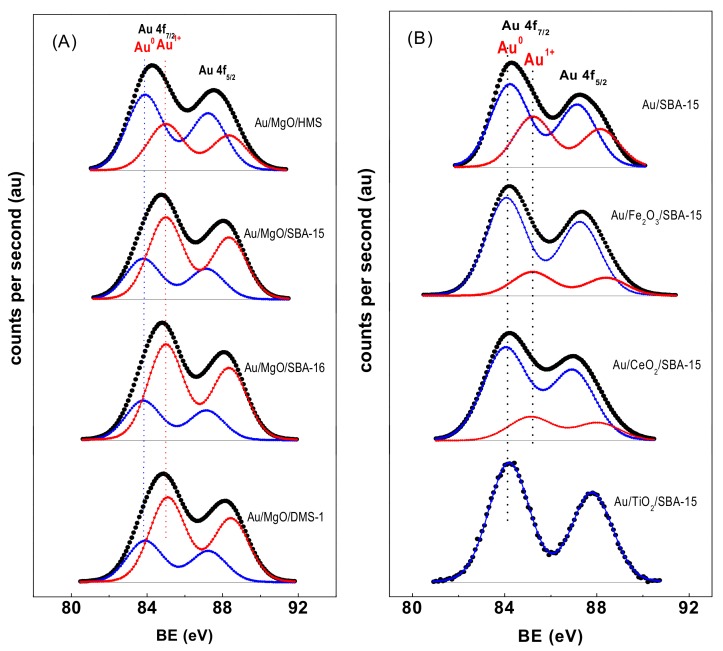
(**A**) XPS curve-fitting of the Au 4f photoelectron peak in the Au catalysts supported on different MSM (**A**) and on SBA-15 modified with reducible metal oxides (**B**).

Further information on the gold oxidation state and its surface exposure in as-prepared samples was obtained by XPS. Figure 8A,B show the Au 4f core level spectra of the Au/MgO/MSM and Au/MO_x_/SBA-15 catalysts, respectively. Binding energy of the core electrons are summarized in Table 3. For all Au catalysts, the Au 4f_7/2_ binding energy (BE) found at 83.8 eV was typical of metallic gold [58], whereas the BE at 85.0 eV was indicative of oxidized surface gold species (Au^1+^) [7]. The presence of both metallic and ionic Au species on the surface of dried catalysts is due to the X-ray beam induced reduction [59]. This well-documented phenomenon [59] was observed also for the dried Au/SiO_2_ catalysts studied by XPS [32]. It is obvious from the data that the Au/TiO_2_/SBA-15 catalyst was the only one showing only metallic Au species. For this catalyst, the O 1 s peak (not shown) showed two components, one small peak at a BE of 530.2 eV due to Ti–O–Ti bonds and the other intense peak at a BE of 532.5 eV, which originated from Si–O–Si linkages. For the Ti 2p_3/2_ core level, the BE value of 458.7 eV was indicative of octahedrally coordinated TiO_2_ species [60,61]. The Fe 2p core-level spectrum (not show) of Au/Fe_2_O_3_/SBA-15 presented the spin-orbit splitting of the Fe 2p_3/2_ ground state at 711.1 eV and the Fe 2p_1/2_ excited state at 724.6 eV. The peak position and energy difference between Fe 2p_3/2_ and Fe 2p_1/2_ (13.5 eV) was typical of the Fe^3+^ state in Fe_2_O_3_ [62].

The surface atomic ratios are summarized in Table 4. Concerning the effect of support structural properties, the comparison of the Au^0^/(Si + M) atomic ratios suggest that the amount of metallic Au species on the surface of HMS-supported catalysts was higher than on the SBA-15, SBA-16, and DMS-1 counterparts, even though these three latter catalysts contain similar Au amounts (Table 2). For all these catalysts, the surface exposure of Au^1+^ species and MO_x_ additives (see M/Si atomic ratio in Table 4) followed the same trend: SBA-15 > SBA-16 > HMS > DMS-1. The preferential location of Au species on MgO rather than on SiO_2_ was expected because the isoelectric point of MgO is higher than that of SiO_2_ [63]. From the apparent lack of peak shape of Au 4f peaks of MO_x_-loaded Au catalysts, and the absence of Au 4f binding energies shift, the Au-support interaction was presumed to be rather weak. This was because the amount of additive was very small as compared to SiO_2_, which is known to exhibit a very weak interaction with Au clusters. On the other hand, it is well-known that binding energy of gold decreases to that of bulk gold as the particle size of the gold cluster increases [64,65]. Thus, the very similar BE values of all samples suggested similar Au particle sizes. 

**Figure 9:**
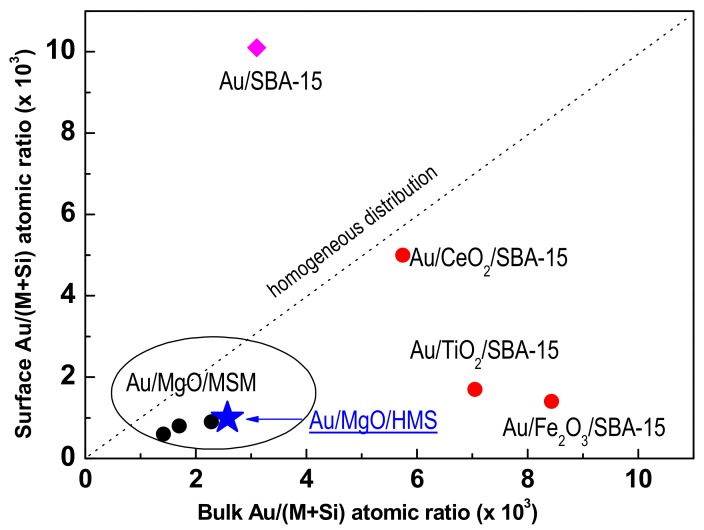
Surface Au/(M + Si) atomic ratio (from XPS) versus bulk Au/(M + Si) atomic ratio (from ICP-OES) of the dried Au catalysts.

To get an idea on the distribution of Au species (either bulk and/or surface), Figure 9 shows the bulk Au/(M + Si) atomic ratio (calculated on the basis of Au loading from ICP-OES) versus surface Au/(M + Si) atomic ratio (from XPS) of the as-prepared Au catalysts. The theoretical value of the homogeneous Au distribution within the samples is represented as a dashed line. Concerning the effect of MO_x_ additive, all samples modified with MgO together with Au/CeO_2_/SBA-15 catalyst possess Au species were more homogeneously dispersed than their counterparts modified with Fe_2_O_3_ and TiO_2_. For all the MgO-modified catalysts, some enrichment in the bulk occurred. For the Au/CeO_2_/SBA-15 catalyst dispersion was similar than for its MgO-loaded counterparts. On the contrary, both the Au/Fe_2_O_3_/SBA-15 and Au/TiO_2_/SBA-15 catalysts showed larger amounts of Au species in the bulk. Contrary to what could be expected, the support morphology had very little influence on the location of Au species, being that the effect of oxide additive was the main factor influencing on the location of Au species. 

### 3.2. Catalytic Activity

In this work, the effects of support structural properties and its modification with metal oxide on the activity of calcined Au catalysts were investigated in CO oxidation at 20 °C carried out in a flow reactor upon atmospheric pressure. For comparison, the catalytic response of two calcined Au/MgO and Au/SBA-15 reference samples was evaluated also. Although the dried Au/TiO_2_ catalyst was reported to be active in CO oxidation, the catalyst activation by calcination led to significant increase of the catalyst activity [66]. In addition, the catalyst calcination demostrated to be more effective than pre-treatment in either a reducing environment or an inert gas [63].

Stoichiometrically, one mole of CO requires half a mole of O_2_ (CO: O_2_ = 1:1/2), but at this ratio, the conversion is usually lower than that desirable, thus, for total CO oxidation excess of oxygen (O_2_/CO ≥ 1.0) is needed [67]. Although there are works using stoichiometric feed [10], activity-time curves bend due to mass transport limitations of oxygen at very high conversions (≥90%). This was the reason, in agreement with bibliography works [66,68,69], why activity tests were conducted using reaction gas composition of 1.0 vol% CO and 1.0 vol% O_2_ (inert gas balance).

**Figure 10:**
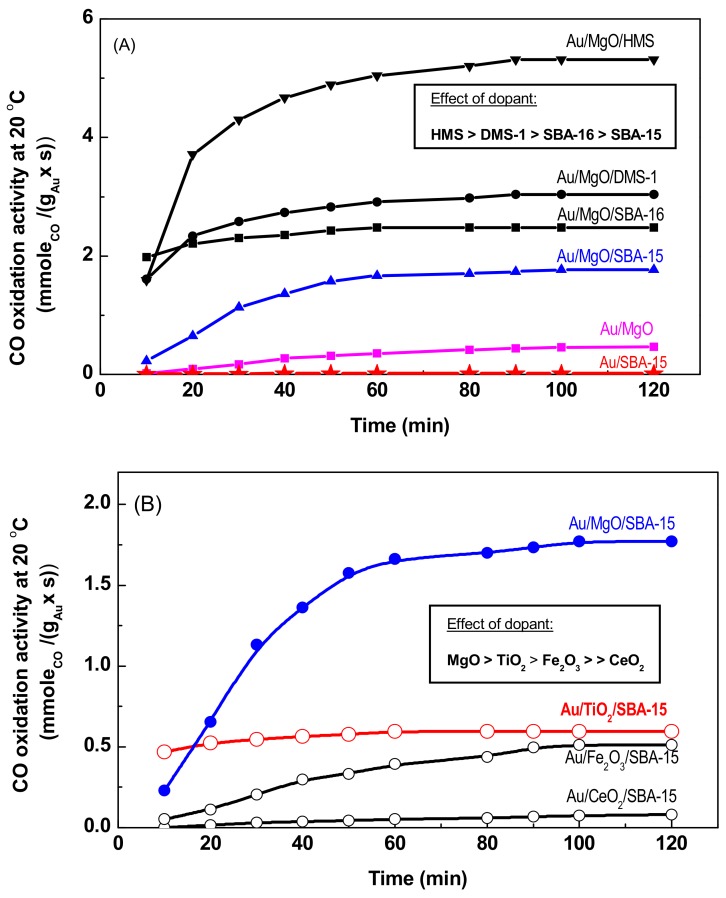
Influence of the support structural properties (**A**) and type of dopant (**B**) on the catalytic response of MSM-based gold catalysts in the total CO oxidation at 20 °C. Gold catalysts were exposed to 1 vol % of CO and 1 vol % of O_2_ (balanced with N_2_ to 1 atm) at 20 °C for 10 min. The Au/MgO and Au/SBA-15 were the reference samples. Solid and open symbols are used for irreducible and reducible dopants, respectively.

Figure 10A shows changes of the mass-specific reaction rates [mole CO/(g_Au_·s)] for the catalysts supported on different substrates modified with MgO. The selected supports were: SBA-16 with 3D cage-like structure, SBA-15 with 2D channel systems, hexagonal mesoporous silica (HMS), and disordered mesoporous silica (DMS-1). Concerning the effect of support structural properties, it was found that catalyst activity at steady-state conditions followed the order: Au/MgO/HMS > Au/MgO/DMS-1 > Au/MgO/SBA-16 > Au/MgO/SBA-15 > Au/MgO/Au/SBA-15 (none). As expected, the Au/SBA-15 reference catalyst was not active upon reaction conditions employed confirming the results reported previously [8,9,14]. The Au/MgO reference sample demonstrated to be less active than all gold catalysts supported on MgO/MSM substrates, which exhibited 100% of CO conversions at the steady-state conditions. From the plots of the specific reaction rates it is inferred that catalyst is activated during time on stream reaction. 

The influence of modifier on catalyst activity is shown in Figure 10B. To obtain differential reaction conditions, the catalysts selected to study of this effect were supported on the SBA-15 carrier (at *TOS* = 10 min, the Au/MgO/SBA-15 exhibit 13% of CO conversion). As shown in Figure 10B, at steady-state conditions, the order of the catalytic activity was as follows: Au/MgO/SBA-15 > Au/TiO_2_/SBA-15 ≈ Au/Fe_2_O_3_/SBA-15 > Au/CeO_2_/SBA-15. The mass-specific reaction rates obtained at steady-state conditions were: 1.77 × 10^−3^, 0.6 × 10^−3^, 0.51 × 10^−3^, and 0.06 × 10^−3^ [mole CO/(g_Au_·s)] for the catalysts modified with MgO, TiO_2_, Fe_2_O_3_, and CeO_2_, respectively. 

**Figure 11:**
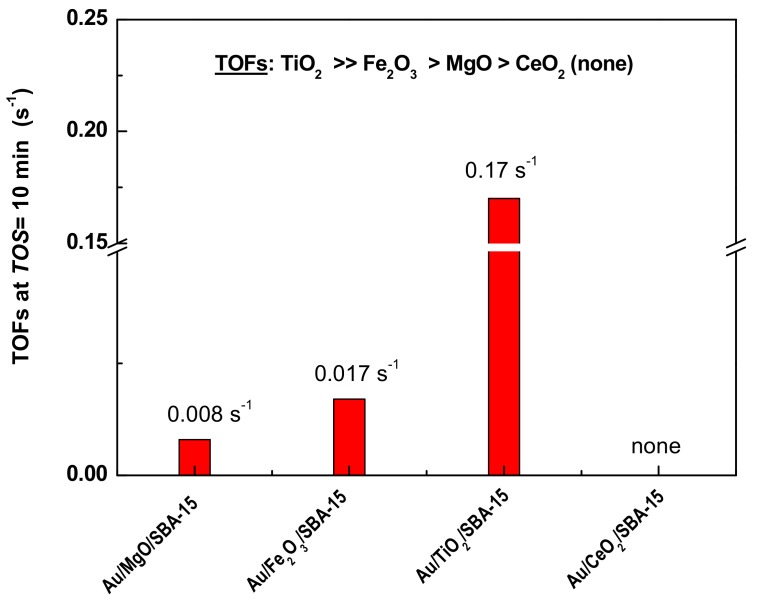
Comparison of the TOFs values of the calcined Au/MO_x_/SBA-15 catalysts tested in the CO oxidation reaction. Reaction conditions were: T = 20 °C, atmospheric pressure, *TOS* = 10 min.

To gain more detailed insight into the influence of modifier on the catalyst initial activity, the turnover frequencies (TOF’s) values were calculated considering initial CO conversion (at *TOS* of 10 min), mean Au particle size (from TEM), and Au loading (Table 2). The comparison of the TOF’s values of the fresh Au/MO_x_/SBA-15 catalysts is shown in Figure 11. As seen, the catalyst intrinsic initial activity followed the trend: Au/TiO_2_/SBA-15 (0.17 s^−1^) >> Au/Fe_2_O_3_/SBA-15 (0.017 s^−1^) > Au/MgO/SBA-15 (0.008 s^−1^) > Au/CeO_2_/SBA-15 (none). This trend clearly indicated that, at the beginning of time course of reaction, the TiO_2_ was a much better modifier than MgO. However, during time course of reaction, this situation changed so that MgO was the best modifier among all the modifiers studied. In addition, it should be clarified that the information obtained from the TOF’s values is limited: This is because the conversion changes during on stream conditions indicating some changes of the active sites dispersion and/or formation of new active phases. In addition, the use of TOFs as the basis for discussing correlations between structure and catalytic activity is fraught with difficulties [68]. This is because of experimental error in the estimation of the number of active sites either by evaluation of the number of surface atoms by selective gas chemisorption [68] or by measurement of the Au particle size by statistical analysis of the particle size from HRTEM images. In the latter case, the hemispherical shape of Au particles is usually assumed. This is not straightforward for all particles, as it was demonstrated by HRTEM of the Au/MO_x_/HMS catalysts (Figure 8) and in many DFT calculations presented in literature [4,38]. 

### 3.3. Discussion

The activity results presented in this work clearly showed that support structural properties together with the type of additive (MO_x_) are two important factors influencing on the final catalytic response of gold catalysts in the low temperature CO oxidation reaction. 

Considering the influence of the support structural properties on the catalyst activity, the key information about the support oxygen storage capacity (OSC) was provided by TPD-O_2_ measurements (Figure 4A). The large difference of the OSC capacities of the naked substrates confirmed that mesoporous silica showed different abilities to reversibly adsorb and store active oxygen. 

The OSC results indicated that the disordered (DMS-1) and wormhole structure (HMS) silica can be more favourable for oxygen storage than the channel-like (SBA-15) and cubic-like (SBA-16) structures. Indeed, the best catalytic response of the calcined Au/MgO/HMS could be explained considering the relatively large oxygen storage capacity of its bare MgO/HMS support (Figure 4). However, taking into account that the largest OSC value of bare SBA-15 and non-reactivity of calcined Au/SBA-15 upon reaction condition used, it is unlikely that the support oxygen storage capacity can be crucial factor for final catalytic response of the silica based system. 

The superior catalytic behaviour of the Au/MgO/HMS catalyst at steady-state conditions can be explained considering its largest amount of active phases and MgO trapped in the pore network, as inferred from its textural properties. In such case, there is a high probability that Au particles can be in close contact with particles of the additive. Taking into account the larger specific BET surface areas of all Au/MgO/MSM catalysts with respect to Au/MgO one (268–574 vs. 17 m^2^ g^−1^) together with the homogeneous dispersion of MgO on the surface of mesoporous silica (from XRD), we conclude that there was a larger perimeter between gold and MgO nanoparticles when Au particles are supported on the MgO/MSM substrates. Indeed, the most active Au/MgO/HMS catalyst exhibited the largest specific BET surface area (574 m^2^ g^−1^) and largest average pore diameter (7.2 nm) among the four MgO-loaded catalysts studied. In addition, both XRD and HRTEM characterization data of the Au/MgO/HMS catalyst indicated that this catalyst showed the lowest crystallite size of Au^0^ phase, which offered a larger amount of Au atoms being in close contact with the dopant. Assuming that the Au cluster binds strongly on MgO(111) with either oxygen or magnesium termination [69], the combined factors of a large oxygen storage capacity of MgO/HMS substrate (Figure 4A) and homogeneous dispersion of Au and MgO nanoparticles (from XRD) may explain the best catalytic response of the Au/MgO/HMS system in the target reaction. In such a case, the dissociation of molecularly adsorbed O_2_ may occur at the metal-additive interface. Atomic oxygen is able to migrate to gold particles via a spill over process [13]. Because the inert character of highly coordinated Au(111) sites as compared with edges and corners [70], a large presence of those defects sites can be inferred for the best catalytic response of Au/MgO/HMS sample.

Concerning the effect of modifier, it was quite clear that highest TOF of Au/TiO_2_/SBA-15 catalyst was due to the exclusive presence of metallic Au species on the surface of this catalyst (from combined DRIFTS-CO and XPS). In addition, the Au/TiO_2_/SBA-15 catalyst was the only one showing the DRIFTS-CO band for step/kink sites (2108 cm^−1^). This strongly suggests that step/kink sites of metallic gold could be responsible for CO oxidation, in line with results observed by Grunwaldt et al. [66]. Taking into account theoretical calculation by Nørskov et al. [38], this can be due to higher adsorption energy of CO molecules on kink or step sites than on terrace sites of Au clusters. It is important to note that the mean size of gold clusters (from both XRD and TEM) in all catalysts were very close to each other (Table 2). Thus, the particle size effect cannot explain the different TOFs values of all SBA-15-based castalysts.

The best catalytic behaviour of MgO-loaded catalysts at steady-state conditions with respect to that modified with reducible oxides strongly suggest that the reducibility of dopant was not the decisive factor influencing on the catalyst activity in the target reaction. This is in line with the study by Comotti et al. [71] who observed that the reducibility of support is not the decisive factor influencing on the catalyst activity in the target reaction. The stabilizing effect of MgO particles on the isolate Au atoms and/or gold clusters on the support surface by vacancies or F-centers and surface OH groups was claimed in literature [72]. With the exception of Au/SBA-15, all catalysts exhibited increases of activity during the time course of the reaction, suggesting improvement of active phase dispersion, development of desirable surface structures on metallic particles (or of perimeter sites between gold and the support), and/or the formation of new cationic gold species [73]. The amount of new active sites formed on the surface of Au/TiO_2_/SBA-15 catalyst should be much lower than that formed on the surface of Au/MgO/SBA-15 catalyst, as deduced from the much larger activation of the latter sample during on-stream conditions. As a consequence, the Au/TiO_2_/SBA-15 catalyst was much less active at steady state conditions than Au/MgO/SBA-15. 

Although the silica modified with metal oxides additives may enhance the metal-support interaction, such interaction is still so weak to prevent sintering of gold particles, as it was confirmed for Au/CeO_2_-SiO_2_ catalysts tested in CO oxidation [15]. Therefore, we assume that an increase of the catalyst activity during on stream reaction can be due to formation of new active sites. Considering the literature findings, the metallic Au is necessary for catalytic activity, but the absence of a correlation between the activity and the extent of reduction implies that cationic Au species may be active sites also [71]. 

**Figure 12:**
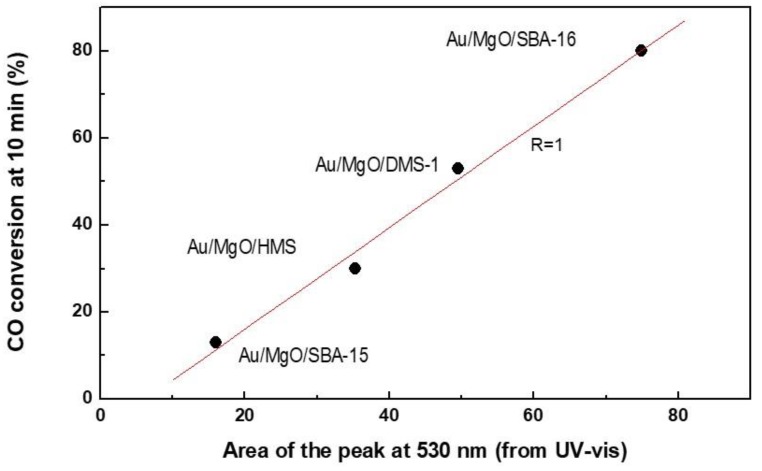
Influence of the surface Au^0^ species (determined by DRIFTS-CO considering area of the peak at 530 nm) on the initial catalyst activity.

Concerning the effect of oxide additive, the best catalytic response of the Au/MgO/SBA-15 catalyst can be explained considering its largest amount of bridged silanol groups suggesting presence of MgO guest molecules within inner support structure. A linear correlation was found between the amount of Au^0^ species, determined by the area of plasmon peak and DRIFTS-CO, and the initial activities of Au/MgO/MSM catalysts (at *TOS* = 10 min) (Figure 12). This correlation strongly suggests that metallic Au species can be in active phases at short reaction times. However, this observation was not general because a similar correlation did not occur for the Au/MO_x_/SBA-15 catalysts, confirming that are various factors influencing on the catalytic response of gold nanoparticles in low temperature CO oxidation. Considering the literature, the main factors influencing on the catalyst behaviour are: (i) specific electronic properties of gold nanoclusters due to quantum-size effect [74], (ii) simultaneous presence of metallic gold and cationic gold species [75], (iii) formation of step sites and defects [6], and (iv) ensemble of Au^0^ atoms and Au cations with hydroxyl ligands of the support [3,5]. For those catalysts, the simultaneous presence of Au^+^ and Au^0^ species (from combined DRIFTS-CO and XPS) suggest that there is some electron transfer from metallic gold particles and the substrate. Thus, although the DRS UV-Vis spectroscopy did not confirm the charge transfer effect between MgO and Au nanoparticles (Figure 6), it may occur because the charge transfer effect between gold and MgO is more significant than in case of gold catalysts supported on SiO_2_ [70]. 

Finally, although there is a large body of work claiming that CeO_2_-supported catalysts are not active in the low temperature CO oxidation [15], our study demonstrated that the Au/CeO_2_/SBA-15 catalyst exhibit some catalytic activity after a large time-on-stream reaction at 20 °C. The lowest activity of the Au/CeO_2/_SBA-15 catalyst among the catalysts studied can be explained, in part, considering the relatively low OSC of the CeO_2_/SBA-15 support (Figure 4B). However, some additional work must be developed to clarify this behaviour.

## 4. Conclusions

In this work, catalytic studies combined with characterization techniques demonstrated the key role of the support structural properties in CO oxidation reaction at 20 °C. The synthetized MSM materials used as supports were: SBA-16 substrate (3D cage-like structure), SBA-15 (2D channel systems), hexagonal mesoporous silica (HMS), and disordered mesoporous silica (DMS-1). 

The best catalytic response was obtained for the HMS-supported catalysts having wormhole structural properties. The superior performance of Au/MgO/HMS catalyst can be explained in terms of multiple factors: (i) the lowest crystal size of Au^0^ particles (from XRD); (ii) the largest reduction degree of Au species (from XPS); (iii) the largest surface exposure of both metallic and cationic gold species on the support surface (from XPS), and (iv) the best textural properties (largest specific BET surface area and main pore diameter).

The MO_x_-free Au/SBA-15 catalyst was not active under the reaction conditions employed (*T* = 20 °C, atmospheric pressure). Surface modification of SBA-15 substrate with reducible oxide additives (Fe_2_O_3_, TiO_2_ and CeO_2_) and non-reducible MgO led to a large enhancement of activity. The best catalytic result was obtained by support modification with MgO additive. This was linked with the highest oxygen storage capacity of the MgO/SBA-15 substrate. 

The oxygen storage capacity of the CeO_2_/SBA-15 substrate was found to be very low compared to the SBA-15 substrate modified with MgO, Fe_2_O_3_ or TiO_2_. As a consequence, the calcined Au/CeO_2_/SBA-15 catalyst exhibited a very low activity in CO oxidation at 20 °C. 

## Figures and Tables

**Table 1:** Metal content ^a^ and textural ^b,c^ properties of the naked supports.

Support	M (wt %)	S_BET_ (m² g^−1^)	NS_BET_	V_total_ (m^3^ g^−1^)	d (nm)
HMS	-	976	-	n.c.	5.6
MgO/HMS	5.2	573	0.6	n.c.	5.7
DMS-1	-	772	-	0.46	2.4
MgO/DMS-1	5.6	165	0.2	0.11	2.8
SBA-16		275	-	0.18	2.6
MgO/SBA-16	5.6	262	1.0	0.18	2.8
SBA-15	-	819	-	0.96	4.7
MgO/SBA-15	5.3	302	0.4	0.43	5.7
Fe_2_O_3_/SBA-15	5.3	634	0.8	0.84	5.7
TiO_2_/SBA-15	7.3	555	0.7	0.69	5.0
CeO_2_/SBA-15	5.6	584	0.8	0.69	4.8

^a^ Metal content (M = Mg, Fe, Ti, Ce) as determined by the ICP-OES technique. ^b^ S_BET_: Brunauer-Emmett-Teller (BET) surface area; V_total_: total volume of pores; d: average pore diameter (calculated from the isotherm adsorption branch) as determined from N_2_ adsorption-desorption isotherms at −196 °C; n.c. = not calculated. ^c^ Normalized S_BET_ of the calculated using the following equation: NS_BET_ = S_BET_ of M_x_O_y_/MSM/[(1 − y) × S_BET_ of MSM] where y is the weight fraction of the modifer (from ICP-OES); S_BET_ of SBA-15 = 819 m^2^/g; M_x_O_y_: MgO; Fe_2_O_3_; CeO_2_, TiO_2_; MSM mesoporous silicas (HMS, SBA-15, SBA-16, DMS-1).

**Table 2:** Gold loading ^a^, textural properties ^b^ and XRD data ^c^ of the dried MSM-supported Au catalysts.

Catalyst	Au ^a^ (wt %)	d_Au_^c^ (nm)	d _MOx_^c^ (nm)	S_BET_ (m² g^−1^)	NS_BET_^d^	V_total_ (m^3^ g^−1^)	d_pores_ (nm)
Au/MgO/HMS	0.9	6.3	n.d.	574	1.0	n.c.	7.2
Au/MgO/DMS-1	0.5	7.4	n.d.	268	1.6	0.27	4.1
Au/MgO/SBA-16	0.6	7.9	n.d.	306	1.2	0.38	4.9
Au/MgO/SBA-15	0.8	7.3 (5.6)	n.d.	398	1.3	0.70	7.0
Au/SBA-15	1.0	n.d. (15.0)	n.d	487	0.6	0.72	5.9
Au/Fe_2_O_3_/SBA-15	2.9	5.9 (7.5)	16.7	467	0.7	0.68	6.3
Au/TiO_2_/SBA-15	2.5	7.6 (5.5)	n.d.	616	1.1	0.81	5.3
Au/CeO_2_/SBA-15	2.7	6.1 (6.2)	4.8	462	0.8	0.67	5.8

^a^ As determined by the ICP-OES technique; ^b^ As determined by determined from N_2_ adsorption-desorption isotherms at −196 °C. ^c^ The crystal size of Au^0^ and MO_X_ phases calculated from the line broadening of the most intense XRD peak using the Debye-Scherrer equation. The Au^0^ crystal size determined by HRTEM is given in parenthesis. n.d. = not detected; n.c. = not calculated. ^d^ Normalized S_BET_ of the calculated using the following equation: NS_BET_ = S_BET_ of catalyst/[(1 − y) × S_BET_ of support] where *y* is the weight fraction of the gold as determined by the ICP-OES technique.

**Table 3:** Binding energies (eV) of core electrons of the dried Au catalysts (from XPS).

Catalyst	Au 4f_7/2_		Fe 2p_3/2_	Mg 2p	Ti 2p_3/2_	Ce 3d_5/2_
	Au^0^	Au^1+^				
Au/MgO/HMS	83.8 (62)	85.1 (38)	-	50.8	-	-
Au/MgO/DMS-1	83.9 (33)	85.1 (67)	-	50.7	-	-
Au/MgO/SBA-16	83.8 (29)	85.0 (71)	-	50.8	-	-
Au/MgO/SBA-15	83.8 (33)	85.0 (67)	-	50.7	-	-
Au/Fe_2_O_3_/SBA-15	83.9 (82)	85.1 (18)	710.7	-	-	-
Au/TiO_2_/SBA-15	84.0 (100)	-	-	-	459.3	-
Au/CeO_2_/SBA-15	83.8 (80)	85.0 (20)	-	-	-	882.8
Au/SBA-15	83.8 (70)	85.0 (30)	-	-	-	-

**Table 4:** Surface atomic ratios of the dried Au catalysts (from XPS).

Catalyst	M/Si ^a^	Au_total_/(Si + M) ^b^ × 10^3^	Au^0^/(Si + M) × 10^3^	Au^1+^/(Si + M) × 10^3^	Au^1+^/Au^0^
Au/MgO/HMS	0.037	1.0 (2.6)	0.6	0.4	0.6
Au/MgO/DMS-1	0.033	0.6 (1.4)	0.2	0.4	2.0
Au/MgO/SBA-16	0.045	0.8 (1.7)	0.2	0.6	2.4
Au/MgO/SBA-15	0.049	0.9 (2.3)	0.3	0.6	2.0
Au/Fe_2_O_3_/SBA-15	0.025	1.4 (8.4)	1.1	0.3	0.3
Au/TiO_2_/SBA-15	0.059	1.7 (7.1)	1.7	-	-
Au/CeO_2_/SBA-15	0.024	5.0 (5.7)	4.0	1.0	0.3
Au/SBA-15	-	10.1 (3.1)	7.1	3.0	0.4

^a^ Surface M/Si atomic ratio (M = Mg, Ce, Fe or Ti). ^b^ Bulk Au/(Si + M) atomic ratios (from ICP-OES) are given in parenthesis.
